# Crystal structure of human serum albumin in complex with megabody reveals unique human and murine cross‐reactive binding site

**DOI:** 10.1002/pro.4887

**Published:** 2024-02-01

**Authors:** Sofia De Felice, Zhanna Romanyuk, Monica Chinellato, Giulia Zoia, Sara Linciano, Yoichi Kumada, Els Pardon, Jan Steyaert, Alessandro Angelini, Laura Cendron

**Affiliations:** ^1^ Department of Biology University of Padua Padua Italy; ^2^ Department of Molecular Sciences and Nanosystems Ca’ Foscari University of Venice Venice Italy; ^3^ Department of Functional Chemistry and Engineering Kyoto Institute of Technology Kyoto Japan; ^4^ VIB‐VUB Center for Structural Biology, VIB Brussels Belgium; ^5^ Structural Biology Brussels, Vrije Universiteit Brussel (VUB) Brussels Belgium; ^6^ European Centre for Living Technology (ECLT), Ca’ Bottacin Venice Italy

**Keywords:** binding mode, cross‐reactivity, drug delivery, megabody, nanobody, pharmacokinetic, serum albumin, yeast surface display

## Abstract

The pharmacokinetic properties of small biotherapeutics can be enhanced via conjugation to cross‐reactive albumin‐binding ligands in a process that improves their safety and accelerates testing through multiple pre‐clinical animal models. In this context, the small and stable heavy‐chain‐only nanobody NbAlb1, capable of binding both human and murine albumin, has recently been successfully applied to improve the stability and prolong the in vivo plasma residence time of multiple small therapeutic candidates. Despite its clinical efficacy, the mechanism of cross‐reactivity of NbAlb1 between human and murine serum albumins has not yet been investigated. To unveil the molecular basis of such an interaction, we solved the crystal structure of human serum albumin (hSA) in complex with NbAlb1. The structure was obtained by harnessing the unique features of a megabody chimeric protein, comprising NbAlb1 grafted onto a modified version of the circularly permutated and bacterial‐derived protein HopQ. This structure showed that NbAlb1 contacts a yet unexplored binding site located in the peripheral region of domain II that is conserved in both human and mouse serum albumin proteins. Furthermore, we show that the binding of NbAlb1 to both serum albumin proteins is retained even at acidic pH levels, thus explaining its extended in vivo half‐life. The elucidation of the molecular basis of NbAlb1 cross‐reactivity to human and murine albumins might guide the design of novel nanobodies with broader reactivity toward a larger panel of serum albumins, thus facilitating the pre‐clinical and clinical phases in humans.

## 1 INTRODUCTION

Human serum albumin (hSA) is a nonglycosylated protein of 66.5 kDa comprising three homologous domains (I, II, and III) that assemble into an asymmetric globular heart‐shape module with up to eight distinct fatty acid–binding sites (Linciano, Moro, et al., 2022). Each domain is divided in two subdomains termed A and B that are composed of four and six α‐helices, respectively (Curry et al., 1998). hSA is characterized by a remarkable solubility, high stability, low immunogenicity, an average concentration in the bloodstream of 600 μM, and a circulatory half‐life of about 3 weeks (Peters, 1996). This long half‐life is mainly related to both its large hydrodynamic diameter (~5 nm) that hinders renal filtration and its ability to bind the neonatal Fc receptor (FcRn) (Sand et al., 2014). The latter mediates the pH‐dependent endocytic recycling of hSA and ultimately its rescue from intracellular lysosomal degradation (Pyzik et al., 2019).

Interestingly, noncovalent binding to hSA sterically shields biotherapeutics, including nucleic acids, peptides, and small proteins, from proteolytic degradation and rapid renal filtration. Moreover, the noncovalent binding allows for reversible detachment of the biotherapeutic from hSA, which facilitates its interaction with the target and improves its penetration and diffusion into regions otherwise inaccessible to large molecules. Overall, these properties make hSA an ideal carrier for enhancing the pharmacokinetic properties and efficacy of numerous biotherapeutics (Zorzi et al., 2019).

As a result, there is a great interest in developing proteins that (i) bind hSA with moderate to high affinity without interfering with its interaction with FcRn, (ii) remain bound to hSA at both neutral and endosomal pH levels, and (iii) can cross‐react with various serum albumins (SAs) derived from different species. Such a system would enhance the speed and safety profile of a biotherapeutic as it progresses through the multiple required pre‐clinical models, ultimately facilitating the advancement to clinical phases in humans (Sand et al., 2014; Zorzi et al., 2019).

Toward this goal, numerous academic research groups and private companies have developed proprietary albumin binders based on the small (~15 kDa) and stable variable domain of the Camelidae heavy–chain‐only (VHH) antibodies, also known as Nanobodies® (Nb) (Muyldermans, 2013; Shen et al., 2021; van Faassen et al., 2020). In particular, one Nb developed by Ablynx, termed NbAlb1, has been reported to bind hSA and its orthologous murine serum albumin (mSA) with affinities of 1.8 and 17.3 nM, respectively (Patent No. US20070269422A1, 2006; Patent No. WO2012175400A1, 2012). Fusing NbAlb1 to a Nb targeting the tumor necrosis factor (TNF) or to a Nb targeting the interleukin‐6 receptor (IL‐6R) led to bispecific biotherapeutics with prolonged half‐lives and superior therapeutic efficacies compared to the monomeric parental anti‐TNF and anti‐IL‐6R Nbs only (Coppieters et al., 2006; Van Roy et al., 2015). Although humanized forms of NbAlb1 are currently in clinical development, there have not been any high‐resolution structural analyses of the interaction between NbAlb1 and hSA.

In the present study, we applied X‐ray crystallography to elucidate the binding mode of NbAlb1 to hSA. Toward this goal, we exploited the unique features of megabodies, consisting of the nanobody grafted in a loop of the circularly permutated *Helicobacter pylori*–derived proteins HopQ or YgjK to provide a higher molecular weight and retained antigen‐binding properties, and explored their versatility beyond single‐particle cryo‐electron microscopy (cryo‐EM) (Uchański et al., 2021). In this work, we observed that NbAlb1 contacts an epitope located in a peripheral region of domain II, which has not yet been explored by other known SA‐binding protein domains. Further binding characterization revealed both that the cross‐reactivity of NbAlb1 is limited to human and murine orthologues and that binding is retained when tested at endosomal pH levels. The identification of the molecular basis of the interaction between NbAlb1 and hSA can serve as a general guideline for the design of novel Nbs with broader cross‐reactivity toward a larger panel of pharmacologically relevant SAs.

## 2 RESULTS AND DISCUSSION

### 2.1 Crystal structure of hSA in complex with megabody

**FIGURE 1:**
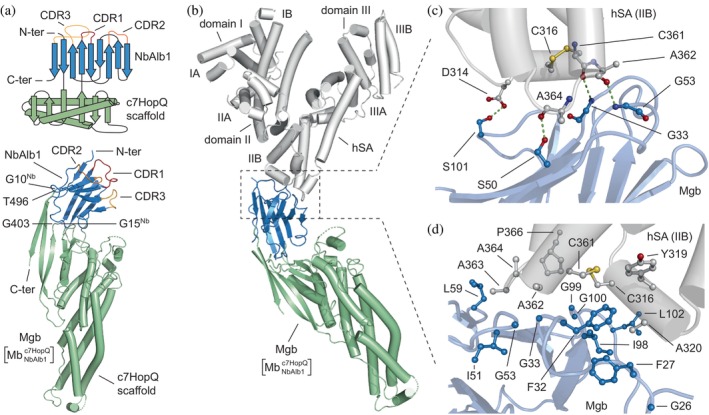
Structure of megabody in complex with hSA. (a) Schematic representation (top) and crystal structure (bottom) of megabody (Mgb) comprising the nanobody Alb1 (NbAlb1; skyblue) grafted into the large circular permutated *H. pylori* adhesin HopQ scaffold protein (c7HopQ; pale green) to obtain ${\mathrm{Mgb}}_{\text{NbAlb}1}^{c7\text{HopQ}}$. The α‐helices are represented as cylinders while the β‐strands are represented as arrows. The complementarity‐determining regions (CDRs) of the nanobody are colored in firebrick (CDR1), bright orange (CDR2), and tv orange (CDR3); (b) Structure of hSA in complex with $Mgb_{\text{NbAlb}1}^{c7\text{HopQ}}$. The structure of hSA (white) is organized in homologous domains (I, II, and III) and subdomains (A and B); (c) Polar interaction network formed by NbAlb1 (skyblue) residues bound to hSA (white); (d) Residues of NbAlb1 (skyblue) and hSA (white) buried and solvent excluded upon complex formation. Selected amino acid side chains are represented as ball‐and‐stick and colored by atom type (hSA: carbon = white, oxygen = firebrick, nitrogen = deep blue; NbAlb1: carbon = sky blue, oxygen = firebrick, nitrogen = deep blue). The three‐dimensional structure was generated and rendered using PYMOL (*The PyMOL Molecular Graphics, Version 2.0; Schrödinger, LLC*., n.d.).

To reveal the binding mode of NbAlb1 to hSA, we determined the three‐dimensional structure via X‐ray crystallography. To stabilize the protein complex, we grafted NbAlb1 onto a large and rigid circular permutated protein derived from *H. pylori* (c7HopQ) to produce a chimeric molecule, called a megabody (Mgb^c7HopQ^_NbAlb1_, henceforth termed Mgb; Figure 1a).

The Mgb‐encoding construct was expressed in *Escherichia coli* and purified to homogeneity by immobilized metal affinity and size exclusion chromatography (SEC). Analytical SEC was also used to confirm the ability of Mgb to bind to hSA and form a stable binary complex (Figure S1b). To stabilize hSA, we carried out co‐crystallization trials in the presence of myristic acid (Myr). The best crystals diffracted to a maximum resolution of 3.30 Å, and the structure of the Mgb in complex with hSA was solved by molecular replacement using a tree‐search method (Table S1, PDB identification code: 8OI2).

In the crystal structure, we were able to trace the polypeptide chain of the Mgb construct from residue 1 to 507, including part of the C‐terminal purification tag. Four connecting loops (52–60, 171–182, 244–251, and 335–346) were not defined since their contribution in the map was uncertain. The crystal structure also revealed that within the Mgb, the NbAlb1 domain adopts a bent orientation if compared to analogous fusion chimeras developed for cryo‐EM studies (Figure 1b; Figure S2) (Uchański et al., 2021), thus proving a certain degree of plasticity of the hinge region connecting c7HopQ and NbAlb1.

We were able to easily trace the electron density of hSA residues for domain II, though it was less defined for the other two domains. In particular, domain I was largely undefined in the experimental maps, and we were unable to detect residues 55–93, 114–124, and 168–177. Indeed, in agreement with previously reported structural studies, hSA reveals a remarkable flexibility in its trilobe architecture. As such, domains not involved in specific ligand interactions exhibit multiple conformations, thus resulting in poorly defined maps (Kovalenko et al., 2013). Regardless, residues that interacted with a ligand were unambiguously defined in the electron density maps, allowing for a clear assignment of their orientations.

In terms of the binding interaction, all three complementarity‐determining regions (CDRs) of NbAlb1 make contacts with hSA, establishing multiple noncovalent interactions with residues located at the peripheral extremity of domain II and covering a total interface surface of 560 Å^2^ (Figure 1c; Tables S1 and S2). More specifically, NbAlb1 interacts with two sides of a groove defined by α‐helix III of hSA subdomain IIB (residues 313–323), a flexible loop (358–P366), and the disulfide bond (C361–C316) that joins the two lobes of the groove (Figure 1b,c). Within this surface, four main hydrogen bonds strengthen the binding of NbAlb1 to hSA. The S101 (CDR3 NbAlb1) forms a hydrogen bond with the carboxylic side chain of D314 (hSA; Figure 1c; Table S2). The backbone nitrogen of G33 (CDR3 NbAlb1) establishes a hydrogen bond with the backbone oxygen of C361 (hSA; Figure 1c; Table S2). The backbone nitrogen of G53 (CDR2 NbAlb1) forms a hydrogen bond with the backbone oxygen of A362 (hSA). Finally, the hydroxyl group of S50 (CDR2 NbAlb1) forms a hydrogen bond with the backbone oxygen of A364 (hSA; Figure 1c; Table S2).

This work also shows that the binding of Mgb to hSA appears to be entropically favored. NbAlb1 binding to hSA promotes the formation of noncovalent interactions that shield the surface of multiple hydrophobic residues of both NbAlb1 (F27, F32, G33, I98, G100, and L106) and hSA (C316, Y319, A320, C361, A362, A363, A364, and P366; Table S3). This shielding contributes to the significant energy gain upon complex formation (−6.8 *K*cal/mol) (Figure 1d). For further details about established contacts and distances, see Table S3. Overall, our structural data revealed that NbAlb1 contacts an epitope located in a peripheral region of domain II, which has not yet been explored by other known SA‐binding protein domains. This interaction occurs via several polar hot‐spots and hydrophobic contacts mediated by all three Nb CDRs.

### 2.2 Nanobody binds an epitope conserved between human and mouse serum albumin

**FIGURE 2:**
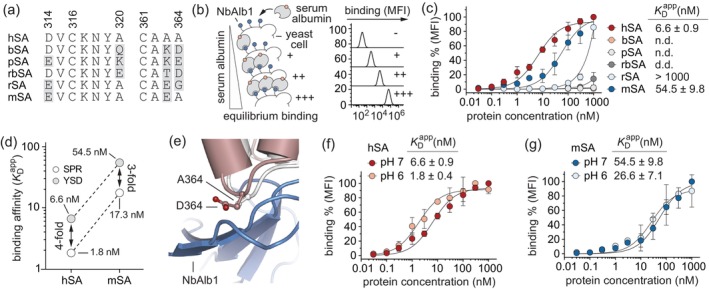
Binding characterization of nanobody Alb1. (a) Alignment of SAs amino acidic residues relevant in the binding interaction with nanobody NbAlb1. Residues that differ from that of hSA sequence are highlighted in grey; (b) Schematic representation of determination of equilibrium dissociation constants (*K*_D_) using yeast surface titration. Yeast cells expressing NbAlb1 are incubated with increasing concentrations of biotinylated SAs. The binding is reported as median fluorescence intensity (MFI) and it is proportional to the amount of SAs bound to the nanobody expressed on the yeast surface; (c) Titrations curves of the equilibrium dissociation constant of NbAlb1 toward multiple SAs determined using yeast surface display (left). The obtained *K*_D_ values expressed in nanomolar range are reported on the right. The plotted values are the results of three independent experiments and are presented as mean (dots) ± s.e.m. (bars); (d) Comparison of the *K*_D_ values of NbAlb1 against hSA and mouse serum albumin (mSA) obtained using two different and complementary techniques: surface plasmon resonance (SPR) and yeast surface display; (e) Superposition of hSA (white) and bovine serum albumin (bSA; dark salmon; PDB identification code: 4F5S) (Bujacz, 2012). Zoom highlighting the importance of residue A364, which is mutated into different amino acids in other investigated SAs, except for mSA, thus explaining its importance in the binding with NbAlb1 (skyblue). Selected amino acid side chains (A364 in hSA and D364 in bSA) are represented as ball‐and‐stick and colored by atom type (carbon = white for hSA and dark salmon for bSA, oxygen = firebrick, nitrogen = deep blue). The three‐dimensional structure was generated and rendered using PYMOL (*The PyMOL Molecular Graphics, Version 2.0; Schrödinger, LLC*., n.d.); (f–g) Titrations curves of the equilibrium dissociation constant of NbAlb1 toward (e) hSA and (f) mSA performed at acidic (pH 6) and physiological (pH 7.4) conditions.

To assess the extent of cross‐reactivity of NbAlb1, we characterized its binding affinity toward multiple orthologue serum albumins (SAs). The tested panel included hSA, bovine SA (bSA), porcine SA (pSA), rabbit SA (rbSA), rat SA (rSA), and murine SA (mSA), the latter of which shares a 72%–76% sequence identity with hSA (Figure 2a). To determine the equilibrium dissociation constant (*K*_D_) of NbAlb1 binding to each SA, we titrated yeast‐displayed NbAlb1 into solutions of the corresponding biotinylated SA (Figure 2b).

These binding studies confirmed the specificity of NbAlb1 for soluble hSA and mSA, with *K*_D_ values of 6.6 and 54.5 nM, respectively. However, weak or no binding was detected toward bSA, pSA, rbSA, and rSA (Figure 2c). These discrepancies in binding affinity observed for the diverse orthologue SAs might be explained by comparing sequences and structures of the contacted epitopes. While hSA and mSA retain an alanine in position 320, bSA, pSA, and rbSA present a bulky and hydrophilic amino acid that might alter the hydrophobic network stabilizing the complex (Figures 1d and 2a). Analogously, both mSA and hSA present an alanine in position 364, which engages in a hydrogen bond with serine in position 50 of NbAlb1 (Figures 1c and 2a,e; Table S2).

Interestingly, the determined *K*_D_ values also averaged 3‐ to 4‐fold higher than those previously reported using surface plasmon resonance (SPR; Figure 2d). This discrepancy might be attributed to the opposite arrangement of the two experiments. Indeed, while yeast surface titrations were performed using soluble biotinylated SAs against the membrane‐anchored NbAlb1, the SPR measurements were executed using a soluble NbAlb1 against chip‐immobilized SAs.

Notably, NbAlb1 could also bind hSA and mSA at both neutral and acidic pH levels (Figure 2f,g). The same behavior has been observed when characterizing the humanized version of NbAlb1, termed NbAlb23 (Figure S2e,f). Thus, the low nanomolar affinity and the ability of NbAlb1 to remain bound to SA throughout the pH gradient of the cellular recycling pathway well explains its extended in vivo half‐life.

### 2.3 Nanobody recognizes an epitope unexplored by other albumin‐binding proteins

**FIGURE 3:**
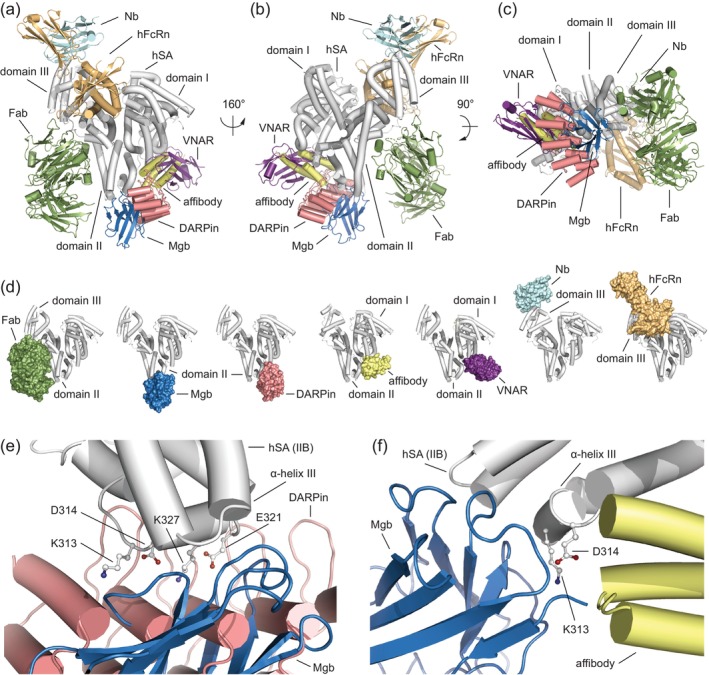
Structural comparison of the ligand binding modes of different albumin‐binding proteins. (a–c) The superimposed hSA‐Mgb (white/sky blue; PDB identification code: 8OI2), hSA‐FcRn (white/light orange; PDB identification code: 4N0F), hSA‐Nb (white/pale cyan; PDB identification code: 5VNW), hSA‐Fab (white/smudge; PDB identification code: 5FUO), hSA‐DARPin (white/salmon; EMDB identification code: 23538); hSA‐affibody (white/pale yellow; PDB identification code: 1TF0) and hSA‐V‐NAR (white/violet purple; PDB identification code: 4HGM) complexes are shown in three orientations (160° and 90° rotations). The α‐helices are represented as cylinders; (d) Molecular surface representation of each single albumin‐binding protein in complex with hSA. From left to right, structure of Fab (smudge) bound to domain II and III of hSA (white), structure of Mgb (sky blue) bound to domain II of hSA (white), structure of DARPin (salmon) bound to domain II of hSA (white), structure of affibody (pale yellow) bound to domain I and II of hSA (white), structure of V‐NAR (violet) bound to domain I and II of hSA (white), structure of Nb (pale cyan) bound to domain III of hSA (white) and structure of FcRn (light orange) bound to domain I and II of hSA (white); (e) Detailed view of the superimposed Mgb (sky blue) and DARPin (salmon) molecules bound domain II of hSA (white). Amino acid side chains of hSA bound by both Mgb and DARPin molecules are represented as ball‐and‐stick and colored by atom type (carbon = white, oxygen = firebrick, nitrogen = deep blue); (f) Detailed view of the superimposed Mgb (sky blue) and affibody (pale yellow) molecules bound domain II of hSA (white). Amino acid side chains of hSA engaged both Mgb and affibody molecules are represented as ball‐and‐stick and colored by atom type (carbon = white, oxygen = firebrick, nitrogen = deep blue). The three‐dimensional structure was generated and rendered using PYMOL (*The PyMOL Molecular Graphics, Version 2.0; Schrödinger, LLC*., n.d.).

We next compared the structure generated in this work with structures of hSA in complex with other albumin‐binding proteins (Figure 3). The other tested molecules were a protein G‐related albumin‐binding (GA) module (Affibody® ABD/GA) (Lejon et al., 2004), an albumin‐binding designed ankyrin repeat protein (DARPin® d2‐21.8.hsa‐c9.v2) (Vulovic et al., 2021), a variable domain derived from cartilaginous fish termed immunoglobulin new antigen receptor (IgNAR; V‐NAR E06) (Kovalenko et al., 2013), a fragment antigen‐binding region of an antibody (Fab CA645) (Adams et al., 2016), a nanobody (Nb.b201) (McMahon et al., 2018), and the human FcRn (Oganesyan et al., 2014).

We then superimposed the crystal structures of hSA in complex with all six above reported protein binders, most of which explored epitopes different from that engaged by NbAlb1 (Figure 3a–c). Indeed, Nb.b201 and the Fab fragment interact with domain III, which is directly involved in the FcRn receptor interaction (Figure 3a–d). The only albumin‐binding proteins that contact hSA domain II near the region occupied by NbAlb1 are DARPin hSA‐c9.v2, affibody ABD/GA, and IgNAR V‐NAR E06 (Figure 3a–d). DARPin hSA‐c9.v2 interacts with hSA mainly via aromatic residues that engage a hydrophobic patch on the surface of hSA. The binding of DARPin also shields residues K313, D314, E321, and K327, which are located in subdomain IIB—right next to those engaged by NbAlb1 (D314, C316, Y319, and A320, Figure 3e). For ABD/GA, the affinity toward hSA is sustained by both hydrophobic interactions with hSA aromatic residues F309 and F326 and polar contacts with K212, N318, and E321. While ABD/GA does interact with domain I and II of hSA, the overlap of surface contacts with NbAlb1 is limited to residues N318 and E321 in α‐helix III of hSA subdomain B (Figure 3f). Overall, our structural comparison revealed that the dorsal surface of subdomain IIB represents a versatile, accessible region that is prone to accommodate multiple ligands. This region could be further explored in the future for the development of binders with broader cross‐reactivity.

## 3 CONCLUSIONS

Our study was the first to examine the binding mechanism of nanobody NbAlb1, revealing that this molecule interacts with an epitope located in subdomain IIB of hSA. This binding occurred at the opposite site to the one engaged by FcRn receptor, thus excluding undesired competition that could impair the physiological recycling of hSA. Furthermore, since the interaction between NbAlb1 and hSA remains unaltered in response to pH changes, NbAlb1 could remain associated with hSA through the endosomal compartment and, in this way, might be rescued from intracellular lysosomal degradation. Altogether, our structural and biochemical data offer an explanation for the superior pharmacokinetic properties previously observed for NbAlb1‐linked biotherapeutics when tested in vivo. Ultimately, our work confirmed that NbAlb1 recognizes an epitope conserved between the distant hSA and mSA proteins, which supports its pharmacological application to only humans and mice. Regardless, the elucidation of the molecular basis of such cross‐reactivity might serve as the foundation for the design of novel nanobodies with broader cross‐reactivity toward a larger panel of SAs derived from different species, thus extending its application to multiple pre‐clinical animal models and ultimately facilitating the transition from pre‐clinical to clinical phases in humans.

## 4 MATERIALS AND METHODS

### 4.1 Cloning, production, and purification of megabody

The megabody design (Mgb; ~55 kDa) was obtained as previously described (Uchański et al., 2021). Mgb consists of a small nanobody (Nb; ~15 kDa) grafted onto the large circular permutated adhesin domain of *H. pylori* (c7HopQ; ~45 kDa) via two short peptide linkers. Bacterial expression vector is based on pMES4 (GenBank GQ907248.1). In brief, the synthetic gene encoding the circular permuted c7HopQ (opened at a surface accessible β‐turn of HopQ and circularly closed via a peptide linker connecting C to N‐terminus) was inserted in the first β‐turn of NbAlb1 to obtain Mgb^c7HopQ^_NbAlb1_ followed by a hexa‐histidine tag (His_6_). Recombinant production of Mb^c7HopQ^_NbAlb1_ fusion protein was performed using *E. coli* WK6 in terrific broth (TB) media. Expression was induced at OD_600_ = 4 by adding 1 mM isopropyl‐β‐d‐1thiogalactopyranoside. Induced cells were maintained at 28°C for 18 h under constant orbital shaking (180 rpm). Cell pellet was collected by centrifugation at 6000 g for 20 min at 4°C and the media was discarded. Cell pellet was resuspended in lysis buffer (30 mM Tris–HCl, 500 mM NaCl, pH 8.5), supplemented with proteases inhibitor cocktail (Abcam, Cambridge, UK) and lysozyme (1 mg mL^−1^, Merck, Darmstadt, Germany), and processed by sonication (Fisher Scientific Model 120 Sonic Dismembrator, Hampton, New Hampshire, USA). Cell debris was harvested by centrifugation at 20000 g for 30 min at 4°C, the supernatant collected, supplemented with 5 mM MgCl_2_, and then incubated with DNaseI 1:10,000 (New England Biolabs, Ipswich, Massachusetts) for 30 min at 4°C. Recombinant Mgb^c7HopQ^_NbAlb1_ protein was purified via immobilized metal affinity chromatography (IMAC) using a HisTrap HP 1 mL column (Cytiva, Freiburg, Germany) connected to an ÄKTA pure FPLC system (Cytiva, Freiburg, Germany) and equilibrated with 30 mM Tris–HCl, 500 mM NaCl, pH 8.5. The resin was then washed extensively using 30 mM Tris–HCl pH 8.5, 150 mM NaCl and eluted in a single peak by applying a linear gradient of imidazole (20–500 mM) at a flow rate of approximately 1 mL min^−1^ in 15 min. Eluted fractions were collected, pooled, concentrated using 10,000 NMWL Amicon Ultra‐15 ultrafiltration devices (Merck, Milan, Italy) at 4000 g and 4°C and further purified via SEC using a HiLoad 10/300 Superdex 200 prep grade column (Cytiva, Freiburg, Germany) connected to an ÄKTA pure FPLC system (Cytiva, Freiburg, Germany) and equilibrated with Tris 30 mM, NaCl 150 mM, pH 8.5. The fractions containing the Mgb^c7HopQ^_NbAlb1_ protein were pooled and further concentrated using 10,000 NMWL Amicon Ultra‐15 ultrafiltration devices (Merck, Darmstadt, Germany) at 4000 g and 4°C to a final protein concentration of 20 mg mL^−1^, determined using a BioPhotometer D30 UV spectrophotometer (Eppendorf, Hamburg, Germany). Purified Mgb^c7HopQ^_NbAlb1_ protein was flash‐frozen in liquid nitrogen and stored at −80°C.

### 4.2 Preparation and purification of recombinant hSA

Recombinant hSA (Albagen XL; UniProt ID: P02768) was purchased from Albumin Bioscience (Alabama, USA) and prepared as previously described (Moro et al., 2022). In brief, the water‐washed activated charcoal (Caesar & Loretz GmbH, Hilden, Germany) was mixed with hSA (0.4 mg charcoal per mg of hSA) initially dissolved in PBS pH 7.4, and the pH was further lowered to three using a 1 M HCl solution. The resulting suspension was incubated for at least 3 h under gentle shaking at 4°C. The pH of the suspension was then adjusted to 7.4 by using a 2 M NaOH solution and filtered using a 0.22 μm membrane filter. The protein aggregates and the disulfide‐bridged dimers formed during this treatment were removed by SEC using a HiLoad 16/600 Superdex 200 prep grade column (Cytiva, Freiburg, Germany) connected to an ÄKTA pure 25 M system (Cytiva, Freiburg, Germany) and equilibrated with 50 mM sodium phosphate buffer (NaPi), 100 mM NaCl, pH 7.4. The fractions containing monomeric delipidated hSA (dhSA) protein were pooled and further concentrated by using 10,000 NMWL Amicon Ultra‐15 ultrafiltration devices (Merck, Milan, Italy) at 4000 g and 4°C on a Heraeus Multifuge X1R centrifuge (Thermo Fisher Scientific, Waltham, MA, USA) to a final protein concentration of 25 mg mL^−1^ (375 μM). Protein concentration was determined using a BioPhotometer D30 UV spectrophotometer (Eppendorf, Hamburg, Germany). Purified dhSA protein was flash‐frozen in liquid nitrogen and stored at −80°C. The monodisperse state of concentrated dhSA protein was confirmed by SEC using a Superdex 200 10/300 GL column (Cytiva, Freiburg, Germany) connected to an ÄKTA pure 25 M system (Cytiva, Freiburg, Germany) and equilibrated with 50 mM NaPi, 100 mM NaCl, pH 7.4. Purified dhSA proteins were eluted as a single peak at elution volume that corresponds to an apparent molecular mass of about 66 kDa (monomer). To stabilize dhSA and homogeneously saturate all its pockets, a re‐lipidation step was performed by incubating dhSA with sodium myristate (Myr; Merck, Milan, Italy), in a 1:10 Myr molar ratio for 1 h at room temperature according to the procedure previously described (Maso et al., 2021).

### 4.3 Complex preparation, characterization, and crystallization

To obtain Mgb^c7HopQ^_NbAlb1_ in complex with lipidated hSA, the two proteins were incubated in equimolar concentrations (30 μM) for 30 min at room temperature. The formation of the complex at physiological conditions (pH 7.4) was confirmed by SEC using a Superdex 200 10/300 GL column (Cytiva, Freiburg, Germany) connected to an ÄKTA pure FPLC system (Cytiva, Freiburg, Germany) and equilibrated with buffer 25 mM Tris, 150 mM NaCl, pH 7.4. Fractions containing Mgb^c7HopQ^_NbAlb1_ in complex with hSA were pooled and further concentrated by using 10,000 MWCO ultrafiltration devices (Merck, Milan, Italy) at 17000 g and 4°C to a final protein concentration of 70 mg mL^−1^. Crystallization trials of Mb^c7HopQ^_NbAlb1_ in complex with hSA were carried out at 285 K in SWISSCI 96‐well 2‐drops MRC crystallization plates (Molecular Dimension Ltd, part of Calibre Scientific, Selden Way, Rotherham, UK) testing the Morpheus and BCS HT‐96 protein crystallization screen kits (Molecular Dimensions Ltd., part of Calibre Scientific, Selden Way, Rotherham, UK). Drops of 0.6 μL volume (0.3 μL of protein complex plus 0.3 μL of reservoir solution) were dispensed using an Oryx 8 crystallization robot (Douglas Instruments Ltd., East Garston, Berkshire, UK) and equilibrated against 80 μL reservoir solution. Plates were sealed and conserved at 20°C. After 1 week, needle‐like crystals were found in B10 Morpheus condition (30 mM sodium fluoride, 30 mM sodium bromide, 30 mM sodium iodide, 100 mM Tris Bicine pH 8.5, 12% v/v ethylene glycol, 6% w/v PEG 8000). Crystals optimization was performed in MRC maxi 48‐well crystallization plates, gradually increasing drop volume from 0.6 to 1.2 μL equilibrated against 120 μL reservoir solution while retaining a 1:1 ratio of protein complex:reservoir. Best crystals were obtained after 1 week in 1.2 μL drops. For X‐ray data collection, crystals were mounted on nylon loops (Hampton Research, Aliso Viejo, CA, USA), soaked for a few seconds in cryoprotectant solution (type B oil), and flash‐frozen in liquid nitrogen.

### 4.4 X‐ray diffraction data collection and processing

X‐ray diffraction data of Mb^c7HopQ^_NbAlb1_ in complex with hSA were collected at ID23‐1 beamline of the European Synchrotron Radiation Facility (ESRF, Grenoble, France). The best crystals of the binary complex diffracted to 3.25 Å maximum resolution. Crystals belonged to the C2 space group, with unit cell parameters: *a* = 370.36 Å, *b* = 73.82 Å, *c* = 66.13 Å, *α* = 90°, *β* = 96.90°, and *γ* = 90°. The asymmetric unit contained one molecule of hSA and Mgb^c7HopQ^_NbAlb1_, corresponding to a Matthews coefficient of 3.75 Å^3^/Da and a solvent content of 67% of the crystal volume. Frames were indexed and integrated with software XIA2, merged, and scaled with AIMLESS (CCP4i2 crystallographic package) (Potterton et al., 2018).

### 4.5 Structure determination and model refinement

The structure was solved by molecular replacement following a tree‐search approach (MOLREP) (Vagin & Teplyakov, 1997). First, we identified a partial solution corresponding to hSA component, using hSA in complex with Myr as a template (PDB identification number: 7AAE) (Maso et al., 2021). Second, we built the Mgb^c7HopQ^_NbAlb1_ structure by modeling it using SWISS‐MODEL server (Waterhouse et al., 2018). The subdomains (c7HopQ and NbAlb1) were either kept as two distinct models or as a circularly permutated fusion construct. The correct solution was obtained starting from the initial partial solution including hSA only and sequentially searching for the additional components NbAlb1 and c7HopQ as two separate entities (Mgb_c7HopQ; PDB identification number: 6XV8) (Uchański et al., 2021). Molecular replacement trials performed using a reconstituted Mgb^c7HopQ^_NbAlb1_ model failed because of the wrongly predicted orientation of NbAlb1 respect to c7HopQ scaffold axis. On the contrary, the multistep method allowed to determine a promising solution that clearly supported the presence of all the three entities (hSA, c7HopQ, and NbAlb1) and allowed the final reconstruction of the full Mgb^c7HopQ^_NbAlb1_ with properly oriented subdomains (Kovalevskiy et al., 2016). The latter has been performed both by manual rebuilding and automated refinement, carried out using LORESTR, PHASER, and REFMAC (McCoy et al., 2007; Vagin et al., 2004). Since the first cycles of refinement, the electron density corresponding to Mgb^c7HopQ^_NbAlb1_ in complex with hSA, especially the hSA domain II directly involved in the interaction, was clearly visible and interpretable in the experimental maps. On the other hand, hSA domain I, that does not establish any relevant crystal contacts neither with Mgb^c7HopQ^_NbAlb1_ nor with other hSA copies, was poorly defined in the electron density maps and thus partially omitted in the final model. Refined structure of Mgb^c7HopQ^_NbAlb1_ in complex with hSA contains 7744 protein atoms and reaches a final crystallographic *R* factor of 0.23 (*R*_free_ 0.28). Intermolecular interactions and the surface involved in the complex definition were analyzed by PISA (Krissinel & Henrick, 2007), LIGPLOT+ (Laskowski & Swindells, 2011), and PYMOL (*The PyMOL Molecular Graphics, Version 2.0; Schrödinger, LLC*., n.d.) software. The structure of Mgb^c7HopQ^_NbAlb1_ in complex with hSA has been deposited in the Protein Data Bank (PDB) with identification code 8OI2. Protein hSA has been numbered from D1 to A585 according to its mature form (lacking the first 24 residues belonging to its secretion signal) (“UniProt: The Universal Protein Knowledgebase in 2021.,” 2021). Mgb^c7HopQ^_NbAlb1_ fusion construct has been deposited following the numbering suggested by Uchański et al., in 2021 (Uchański et al., 2021), while in the Results and Discussion section, standard IMGT Nbs numbering system has been adopted to realign the discussion to standard CDRs assignment and allow a direct comparison with other Nbs.

### 4.6 Chemical biotinylation of serum albumin proteins

Recombinant bovine serum albumin (bSA, UniProt ID: P02769), rabbit serum albumin (rbSA, UniProt ID: P49065), pig serum albumin (pSA, UniProt ID: P08835), and rat serum albumin (rSA, UniProt ID: P02770) were purchased from Sigma‐Aldrich (Milan, Italy). Recombinant mouse serum albumin (mSA, UniProt ID: P07724) was purchased from Albumin Bioscience (Huntsville, AL, USA). Serum albumin proteins were buffer exchanged by SEC using HiLoad 16/600 Superdex 200 prep grade column (Cytiva, Freiburg, Germany) connected to an AKTApure 25 M system (Cytiva, Freiburg, Germany) and equilibrated with buffer PBS pH 7.4. Fractions containing monomeric serum albumin protein were pooled and further concentrated by using 10,000 NMWL Amicon Ultra‐15 ultrafiltration devices (Merck, Milan, Italy) at 4000 g and 4°C to a final protein concentration of 2 mg mL^−1^ (30 μM). Reactive EZ‐link sulfo‐NHS‐LC‐biotin (Thermo Fisher Scientific, Waltham, MA, USA) was dissolved in PBS pH 7.4 to obtain a final concentration of 10 mM. Serum albumin conjugates containing biotin were prepared by incubating serum albumin proteins (at concentrations of 2 mg mL^−1^ in PBS pH 7.4) with 10‐fold molar excess of EZ‐link sulfo‐NHS‐LC‐biotin for 60 min at room temperature. Excess of unreacted or hydrolyzed biotinylation reagent was removed using a High prep 26/10 desalting column (Cytiva, Freiburg, Germany) connected to an AKTApure 25 M system (Cytiva, Freiburg, Germany) and equilibrated with buffer PBS pH 7.4. Fractions corresponding to the expected protein peak were pooled and concentrated using 10,000 NMWL Amicon Ultra‐4 ultrafiltration devices (Merck, Milan, Italy) at 4000 g and 4°C. Final protein concentrations were measured using a BioPhotometer D30 UV spectrophotometer (Eppendorf, Hamburg, Germany).

### 4.7 Display of nanobody on the surface of yeast cells

The nanobody NbAlb1 was displayed on the surface of yeast as N‐terminal fusion of a flexible stalk region anchored to the yeast cell wall (Linciano et al., 2019). The synthetic DNA coding for NbAlb1 was obtained from GeneArt Gene Synthesis (Thermo Fisher Scientific, Waltham, MA, USA). Gene was codon‐optimized for expression in yeast cells. The de novo synthesized gene was PCR amplified using Phusion high‐fidelity polymerase (Thermo Fisher Scientific, Waltham, MA, USA) and the following oligonucleotide primers (5′‐GGCTGAGGCTGCTAGCGCTGTTCAATTGGTTGAATCCGGTG‐3′, 5′‐TGAGATCCGGATCCAGAAGAAACAGTAACCTGAGTACCCTG‐3′) from Integrated DNA Technologies (Integrated DNA Technologies, Coralville, USA). The gene was subsequently subcloned into an in‐house modified yeast‐display vector via *Nhe*I and *Bam*HI restriction enzymes (New England Lab, Ipswich, USA). The final construct included a DNA sequence encoding for a αmating factor secretory leader sequence, the NbAlb1 gene, a 15 amino acid flexible spacer (^N^GGGGSGGGGSGGGGS^C^), a sequence encoding for the influenza hemagglutinin epitope tag (HA; ^N^YPYDVPDYA^C^) followed by a sequence encoding for the anchor protein 649‐stalk to obtain ^N^NbAlb1‐(G_4_S)_3_‐HA‐pStalk^C^ fusion protein. Construct was verified by DNA sequencing. Genetically modified *Saccharomyces cerevisiae* yeast cells (EBY100 strain) were transformed with DNA plasmid encoding ^N^NbAlb1‐(G_4_S)3‐HA‐pStalk^C^ fusion protein using Frozen‐EZ Yeast Transformation II Kit (Zymo Research, Orange, USA) and plated on selective SD‐CAA solid agar media. Single colony was picked and inoculated in 5 mL SD‐SCAA culture, grown to mid‐log phase (OD_600_ = 2–5) in SD‐CAA media at 30°C with shaking (250 rpm) and induced in SG‐CAA media for 20 h at 20°C with shaking (250 rpm) (Angelini et al., 2015). Staining of C‐terminus HA epitope tag indicated that ^N^NbAlb1‐(G_4_S)_3_‐HA‐pStalk^C^ was expressed well on the surface of yeast.

### 4.8 Determination of equilibrium dissociation constants using yeast surface titration

The equilibrium dissociation constant (*K*_D_) of nanobody NbAlb1 toward multiple serum albumins was determined using yeast surface display titrations (Angelini et al., 2018; Linciano, Wong, et al., 2022). The binding assays were conducted in 96‐well plates (VWR, Radnor, PA, USA) containing 2 × 10^5^ induced yeast cells per well. Yeast cells displaying NbAlb1 were incubated with varying concentrations of multiple soluble biotinylated SAs and the primary mouse anti‐HA epitope tag antibody (clone 2–2.214, 1:1000 dilution; Thermo Fisher Scientific, Waltham, MA, USA) for 1 h at 4°C with shaking (150 rpm). Ten different concentrations of pure biotinylated SAs, ranging from 30 pM to 1 μM, were applied. After primary incubation, cells were pelleted (2500 g for 5 min at 4°C) and washed twice with 200 μL ice‐cold PBS buffer at pH 7.4 supplemented with 0.01% w/v casein. Secondary labeling was performed with goat anti‐mouse IgG conjugated to DyLight™ 488 (1:500 dilution, Thermo Fisher Scientific, Waltham, MA, USA) and NeutrAvidin™ conjugated to DyLight™ 650 (1:500 dilution, Thermo Fisher Scientific, Waltham, MA, USA). The binding assays were performed also in acidic conditions, using a 25 mM MES, 150 mM NaCl at pH 6 buffer supplemented with 0.01% w/v casein. The 96‐well plates were run on a high throughput plate sampler CytKick MAX Auto Sampler (Thermo Fisher Scientific, Waltham, MA, USA) or individually analyzed on an Attune NxT Acoustic Focusing Cytometer (Thermo Fisher Scientific, Waltham, MA, USA). Data were evaluated using FlowJo v.10.6.2 software. The median fluorescence intensity (MFI) from binding signal as a function of protein concentration was used to determine the *K*_D_ values for all SAs of interest. Values reported are the results of three independent experiments and are presented as mean (dots) ± s.e.m. (bars).

## AUTHOR CONTRIBUTIONS

**Laura Cendron:** Conceptualization; writing – original draft; methodology; writing – review and editing; project administration; resources; supervision; validation. **Sofia De Felice:** Conceptualization; formal analysis; methodology; writing – original draft; investigation; data curation; writing – review and editing. **Zhanna Romanyuk:** Investigation; methodology; data curation; writing – original draft; formal analysis; visualization. **Monica Chinellato:** Investigation; formal analysis; methodology; writing – original draft. **Giulia Zoia:** Investigation; formal analysis; data curation; methodology. **Sara Linciano:** Investigation; writing – review and editing. **Yoichi Kumada:** Resources; supervision; writing – review and editing. **Els Pardon:** Writing – review and editing; methodology; investigation; validation. **Jan Steyaert:** Resources; writing – review and editing. **Alessandro Angelini:** Resources; supervision; conceptualization; project administration; writing – original draft; writing – review and editing; validation.

## CONFLICT OF INTEREST STATEMENT

The authors declare that the research was conducted in the absence of any commercial or financial relationships that could be construed as a potential conflict of interest.

## Supporting information


Data S1.

## Data Availability

Atomic coordinates and structure factors were deposited into the Protein Data Bank under the accession code 8OI2.

## References

[pro4887-bib-0001] Adams R , Griffin L , Compson JE , Jairaj M , Baker T , Ceska T , et al. Extending the half‐life of a fab fragment through generation of a humanized anti‐human serum albumin Fv domain: an investigation into the correlation between affinity and serum half‐life. MAbs. 2016;8(7):1336–1346. 10.1080/19420862.2016.1185581 27315033 PMC5058626

[pro4887-bib-0002] Angelini A , Chen TF , de Picciotto S , Yang NJ , Tzeng A , Santos MS , et al. Protein engineering and selection using yeast surface display. Methods Mol Biol. 2015;1319:3–36. 10.1007/978-1-4939-2748-7_1 26060067

[pro4887-bib-0003] Angelini A , Miyabe Y , Newsted D , Kwan BH , Miyabe C , Kelly RL , et al. Directed evolution of broadly crossreactive chemokine‐blocking antibodies efficacious in arthritis. Nat Commun. 2018;9(1): 1461. 10.1038/S41467-018-03687-X PMC5899157 29654232

[pro4887-bib-0004] Beirnaert E , Revets HAP , Hoogenboom HRJM , Jonckheere HMF , Dreier T . Patent No. US20070269422A1. Serum albumin binding proteins with long half‐lives. 2006.

[pro4887-bib-0005] Bujacz A . Structures of bovine, equine and leporine serum albumin. Acta Crystallogr D Biol Crystallogr. 2012;68(Pt 10):1278–1289. 10.1107/S0907444912027047 22993082

[pro4887-bib-0006] Coppieters K , Dreier T , Silence K , de Haard H , Lauwereys M , Casteels P , et al. Formatted anti‐tumor necrosis factor alpha VHH proteins derived from camelids show superior potency and targeting to inflamed joints in a murine model of collagen‐induced arthritis. Arthritis Rheum. 2006;54(6):1856–1866. 10.1002/art.21827 16736523

[pro4887-bib-0007] Curry S , Mandelkow H , Brick P , Franks N . Crystal structure of human serum albumin complexed with fatty acid reveals an asymmetric distribution of binding sites. Nat Struct Biol. 1998;5(9):827–835. 10.1038/1869 9731778

[pro4887-bib-0008] Dombrecht B , Peter S , Ververken CJN . Patent No. WO2012175400A1. Serum albumin binding proteins. 2012.

[pro4887-bib-0009] Kovalenko OV , Olland A , Piché‐Nicholas N , Godbole A , King D , Svenson K , et al. Atypical antigen recognition mode of a shark immunoglobulin new antigen receptor (IgNAR) variable domain characterized by humanization and structural analysis. J Biol Chem. 2013;288(24):17408–17419. 10.1074/jbc.M112.435289 23632026 PMC3682541

[pro4887-bib-0010] Kovalevskiy O , Nicholls RA , Murshudov GN . Automated refinement of macromolecular structures at low resolution using prior information. Acta Crystallogr D Struct Biol. 2016;72(Pt 10):1149–1161. 10.1107/S2059798316014534 27710936 PMC5053141

[pro4887-bib-0011] Krissinel E , Henrick K . Inference of macromolecular assemblies from crystalline state. J Mol Biol. 2007;372(3):774–797. 10.1016/j.jmb.2007.05.022 17681537

[pro4887-bib-0012] Laskowski RA , Swindells MB . LigPlot+: multiple ligand‐protein interaction diagrams for drug discovery. J Chem Inf Model. 2011;51(10):2778–2786. 10.1021/ci200227u 21919503

[pro4887-bib-0013] Lejon S , Frick I‐M , Björck L , Wikström M , Svensson S . Crystal structure and biological implications of a bacterial albumin binding module in complex with human serum albumin. J Biol Chem. 2004;279(41):42924–42928. 10.1074/jbc.M406957200 15269208

[pro4887-bib-0014] Linciano S , Moro G , Zorzi A , Angelini A . Molecular analysis and therapeutic applications of human serum albumin‐fatty acid interactions. J Control Release. 2022;348:115–126. 10.1016/j.jconrel.2022.05.038 35643382

[pro4887-bib-0015] Linciano S , Pluda S , Bacchin A , Angelini A . Molecular evolution of peptides by yeast surface display technology. MedChemComm. 2019;10(9):1569–1580. 10.1039/c9md00252a 31803399 PMC6836575

[pro4887-bib-0016] Linciano S , Wong EL , Mazzocato Y , Chinellato M , Scaravetti T , Caregnato A , et al. Guidelines, strategies, and principles for the directed evolution of cross‐reactive antibodies using yeast surface display technology. Methods Mol Biol. 2022;2491:251–262. 10.1007/978-1-0716-2285-8_14 35482195

[pro4887-bib-0017] Maso L , Trande M , Liberi S , Moro G , Daems E , Linciano S , et al. Unveiling the binding mode of perfluorooctanoic acid to human serum albumin. Protein Sci. 2021;30(4):830–841. 10.1002/pro.4036 33550662 PMC7980502

[pro4887-bib-0018] McCoy AJ , Grosse‐Kunstleve RW , Adams PD , Winn MD , Storoni LC , Read RJ . Phaser crystallographic software. J Appl Crystallogr. 2007;40(Pt 4):658–674. 10.1107/S0021889807021206 19461840 PMC2483472

[pro4887-bib-0019] McMahon C , Baier AS , Pascolutti R , Wegrecki M , Zheng S , Ong JX , et al. Yeast surface display platform for rapid discovery of conformationally selective nanobodies. Nat Struct Mol Biol. 2018;25(3):289–296. 10.1038/s41594-018-0028-6 29434346 PMC5839991

[pro4887-bib-0020] Moro G , Liberi S , Vascon F , Linciano S , De Felice S , Fasolato S , et al. Investigation of the interaction between human serum albumin and branched short‐chain perfluoroalkyl compounds. Chem Res Toxicol. 2022;35(11):2049–2058. 10.1021/acs.chemrestox.2c00211 36148994 PMC9682524

[pro4887-bib-0021] Muyldermans S . Nanobodies: natural single‐domain antibodies. Annu Rev Biochem. 2013;82:775–797. 10.1146/annurev-biochem-063011-092449 23495938

[pro4887-bib-0022] Oganesyan V , Damschroder MM , Cook KE , Li Q , Gao C , Wu H , et al. Structural insights into neonatal fc receptor‐based recycling mechanisms. J Biol Chem. 2014;289(11):7812–7824. 10.1074/jbc.M113.537563 24469444 PMC3953293

[pro4887-bib-0023] Peters TJ . All about albumin: biochemistry, genetics and medical applications. Diego, CA: Academic Press Inc; 1996.

[pro4887-bib-0024] Potterton L , Agirre J , Ballard C , Cowtan K , Dodson E , Evans PR , et al. CCP4i2: the new graphical user interface to the CCP4 program suite. Acta Crystallogr D Struct Biol. 2018;74(Pt 2):68–84. 10.1107/S2059798317016035 29533233 PMC5947771

[pro4887-bib-0025] Pyzik M , Sand KMK , Hubbard JJ , Andersen JT , Sandlie I , Blumberg RS . The neonatal fc receptor (FcRn): a misnomer? Front Immunol. 2019;10:1540. 10.3389/fimmu.2019.01540 31354709 PMC6636548

[pro4887-bib-0026] Sand KMK , Bern M , Nilsen J , Noordzij HT , Sandlie I , Andersen JT . Unraveling the interaction between FcRn and albumin: opportunities for Design of Albumin‐Based Therapeutics. Front Immunol. 2014;5:682. 10.3389/fimmu.2014.00682 25674083 PMC4306297

[pro4887-bib-0027] Shen Z , Xiang Y , Vergara S , Chen A , Xiao Z , Santiago U , et al. A resource of high‐quality and versatile nanobodies for drug delivery. IScience. 2021;24(9):103014. 10.1016/j.isci.2021.103014 34522857 PMC8426283

[pro4887-bib-0028] The PyMOL Molecular Graphics, Version 2.0. Schrödinger, LLC; n.d. 2015; New York.

[pro4887-bib-0029] Uchański T , Masiulis S , Fischer B , Kalichuk V , López‐Sánchez U , Zarkadas E , et al. Megabodies expand the nanobody toolkit for protein structure determination by single‐particle cryo‐EM. Nat Methods. 2021;18(1):60–68. 10.1038/s41592-020-01001-6 33408403 PMC7611088

[pro4887-bib-0030] UniProt Consortium . UniProt: the universal protein knowledgebase in 2021. Nucleic Acids Res. 2021;49(D1):D480–D489. 10.1093/nar/gkaa1100 33237286 PMC7778908

[pro4887-bib-0031] Vagin A , Teplyakov A . MOLREP: an automated program for molecular replacement. J Appl Crystallogr. 1997;30(6):1022–1025. 10.1107/S0021889897006766

[pro4887-bib-0032] Vagin AA , Steiner RA , Lebedev AA , Potterton L , McNicholas S , Long F , et al. REFMAC5 dictionary: organization of prior chemical knowledge and guidelines for its use. Acta Crystallogr D Biol Crystallogr. 2004;60(Pt 12 Pt 1):2184–2195. 10.1107/S0907444904023510 15572771

[pro4887-bib-0033] van Faassen H , Ryan S , Henry KA , Raphael S , Yang Q , Rossotti MA , et al. Serum albumin‐binding V(H) Hs with variable pH sensitivities enable tailored half‐life extension of biologics. FASEB J. 2020;34(6):8155–8171. 10.1096/fj.201903231R 32342547

[pro4887-bib-0034] Van Roy M , Ververken C , Beirnaert E , Hoefman S , Kolkman J , Vierboom M , et al. The preclinical pharmacology of the high affinity anti‐IL‐6R nanobody® ALX‐0061 supports its clinical development in rheumatoid arthritis. Arthritis Res Ther. 2015;17(1):135. 10.1186/s13075-015-0651-0 25994180 PMC4476083

[pro4887-bib-0035] Vulovic I , Yao Q , Park Y‐J , Courbet A , Norris A , Busch F , et al. Generation of ordered protein assemblies using rigid three‐body fusion. Proc Natl Acad Sci U S A. 2021;118(23). 10.1073/pnas.2015037118 PMC8201882 34074752

[pro4887-bib-0036] Waterhouse A , Bertoni M , Bienert S , Studer G , Tauriello G , Gumienny R , et al. SWISS‐MODEL: homology modelling of protein structures and complexes. Nucleic Acids Res. 2018;46(W1):W296–W303. 10.1093/nar/gky427 29788355 PMC6030848

[pro4887-bib-0037] Zorzi A , Linciano S , Angelini A . Non‐covalent albumin‐binding ligands for extending the circulating half‐life of small biotherapeutics. MedChemComm. 2019;10(7):1068–1081. 10.1039/c9md00018f 31391879 PMC6644573

