# Supplementary material for: Crystal structure of human serum albumin in complex with megabody reveals unique human and murine cross‐reactive binding site
Source: Protein Sci. 2024 Feb 1;33(2):e4887. doi: 10.1002/pro.4887 (PMC10804666; doi:10.1002/pro.4887)
Supplement: Supplementary file 1 — Data S1. [file PRO-33-e4887-s001.docx]

**Crystal structure of human serum albumin in complex with megabody reveals unique human and murine cross-reactive binding site**

Sofia De Felice^1^, Zhanna Romanyuk^2^, Monica Chinellato^1^, Giulia Zoia^2^, Sara Linciano^2^, Yoichi Kumada^3^, Els Pardon^4,5^, Jan Steyaert^4,5^, Alessandro Angelini^2,6*^ and Laura Cendron^1*^

^1^ Department of Biology, University of Padua, Viale G. Colombo 3, 35131 Padua, Italy

^2^ Department of Molecular Sciences and Nanosystems, Ca’ Foscari University of Venice, Via Torino 155, 30172 Venice, Italy

^3^ Department of Functional Chemistry and Engineering, Kyoto Institute of Technology, 1 Matsugasaki-Hashikami-Cho, Sakyo-ku, Kyoto 606-0951, Japan

^4^ VIB-VUB Center for Structural Biology, Vrije Universiteit Brussel, Pleinlaan 2, 1050 Brussels, Belgium

^5^ Structural Biology Brussels, Vrije Universiteit Brussel (VUB), Pleinlaan 2, 1050 Brussels, Belgium

^6^ European Centre for Living Technology (ECLT), Ca’ Bottacin, Dorsoduro 3911, Calle Crosera, 30123 Venice, Italy

**Supplementary Tables**

| Data collection * |  |
| --- | --- |
| Beamline | ID 23-1 |
| Wavelength (Å) | 0.8856 |
| Space group | C 1 2 1 |
| **Cell parameters** |  |
| a, b, c (Å); α, β, γ (°) | 370.36, 73.82, 66.13, 90.00, 96.90, 90.00 |
| Resolution (Å) | 34.25 – 3.25 (3.53 – 3.30) |
| Unique observations | 26536 (4847) |
| Multiplicity | 3.0 (3.0) |
| R_merge_ | 0.044 (0.937) |
| <I / σ(I)> | 11.0 (1.1) |
| CC1/2 | 0.998 (0.480) |
| Completeness (%) | 98.4 (99.6) |
| **Refinement** |  |
| No. reflections (used for R_free_ calculation) | 25728 (2577) |
| R_work_ / R_free_ | 0.226/0.274 |
| Number non-hydrogen atoms | 7692 |
| protein | 7692 |
| ligands | 0 |
| solvent | 0 |
| Others (EDO, MPD, MOPS, PG4, PGE) | 0 |
| **Geometry** |  |
| RMSD values |  |
| bond lengths (Å) | 0.004 |
| bond angles (°) | 0.68 |
| Ramachandran plot (%) |  |
| most favoured | 93.17 |
| additionally allowed | 6.0 |
| outliers | 0.83 |
| Rotamers outliers (%) | 1.89 |
| Average B-factor | 217.13 |

**Supplementary table 1**. Statistics on X-ray diffraction data collection and refinement. Frames were measured in 0.1° oscillation steps at 100 K. A single crystal was used to collect all diffraction data. Highest-resolution shell statistics are shown within brackets.

| **hSA atom / residue** | | **NbAlb1 atom / residue** | **Distance** (Å) |
| --- | --- | --- | --- |
| OD1 / Asp314 | N / Ser101 (488) [CDR2] | | 3.54 |
| O / Cys361 | N / Gly33 (420) [CDR1] | | 2.74 |
| O / Ala362 | N / Gly53 (440) [CDR3] | | 2.74 |
| O / Ala364 | OG100 /Ser50 (437) [CDR3] | | 2.67 |

**Supplementary table 2**. Atoms and residues engaged in hydrogen bonds between hSA and NbAlb1 are reported. Numbers referring to Mgb are shown in round brackets. The complementary determining regions (CDRs) of the NbAlb1 to which the residues involved in the interaction belongs are indicated in square brackets. Hydrogen bonds (HB) were defined using the web server PISA (Krissinel & Henrick, 2007).

| **hSA atom / residue** | | **NbAlb1 atom / residue** | **Distance** (Å) |
| --- | --- | --- | --- |
| OD1 / Asp314 | CA / Gly100 (487) | | 3.28 |
| OD1 / Asp314 | C / Gly100 (487) | | 3.81 |
| OD2 / Asp314 | CB / Leu102 (489) | | 3.73 |
| O / Cys316 | CE1 / Phe32 (419) | | 3.26 |
| NZ / Lys317 | CG / Arg104 (491) | | 3.33 |
| N / Ala320 | CZ / Gly100 (487) | | 3.79 |
| CA / Ala320 | CZ / Gly100 (487) | | 3.53 |
| CB / Ala320 | CE1 / Gly100 (487) | | 3.56 |
| CB / Ala320 | CE2 / Gly100 (487) | | 3.75 |
| CB / Ala320 | CZ / Gly100 (487) | | 3.23 |
| OE2 / Glu321 | CG2 / Val 2 (2) | | 3.89 |
| C / Cys361 | N / Gly33 (420) | | 3.82 |
| O / Cys361 | CA / Phe32 (419) | | 3.45 |
| O / Cys361 | C / Phe32 (419) | | 3.56 |
| O / Cys361 | CA / Gly33 (420) | | 3.70 |
| N / Ala362 | O / Ser31 (418) | | 3.80 |
| CA / Ala362 | O / Ser31 (418) | | 3.57 |
| CA / Ala362 | N / Gly33 (420) | | 3.76 |
| C / Ala362 | N / Gly53 (440) | | 3.64 |
| O / Ala362 | CA / Ser52 (439) | | 3.74 |
| O / Ala362 | C / Ser52 (439) | | 3.70 |
| O / Ala362 | CB / Ser52 (439) | | 3.59 |
| O / Ala362 | CA / Gly53 (440) | | 3.53 |
| CB / Ala362 | C / Ser31 (418) | | 3.77 |
| CB / Ala362 | O / Ser31 (418) | | 3.77 |
| C / Ala363 | CA / Ser52 (439) | | 3.35 |
| C / Ala363 | CB / Ser52 (439) | | 3.35 |
| O / Ala363 | CA / Gly33 (420) | | 3.55 |
| N / Ala364 | CA / Ser52 (439) | | 3.64 |
| N / Ala364 | CB / Ser52 (439) | | 3.53 |
| CA / Ala364 | C / Ile51 (438) | | 3.90 |
| CA/Ala364 | N/Ser52 (439) | | 3.79 |
| CA/Ala364 | CA/Ser52 (439) | | 3.83 |
| C/Ala364 | OG/Ser50 (437) | | 3.67 |
| O/Ala364 | CB/Ser 50 (437) | | 3.79 |
| CB/Ala364 | N/Ser52 (439) | | 3.71 |
| CB/Ala364 | O/Asp57 (444) | | 3.71 |
| CB/Ala364 | CB/Asp57 (444) | | 3.72 |
| CB/Ala364 | CD2/Leu59 (446) | | 3.78 |

**Supplementary table 3.** Non bonded contacts established by NbAlb1 (Mgb) residues and hSA- upon interaction. Residues numbers referring to Mgb are shown in round brackets. The complementary determining regions (CDRs) of the NbAlb1 to which the residues involved in the interaction belongs are indicated in square brackets. Interactions analysis has been performed with PISA and PDBsum severs (Krissinel & Henrick, 2007) (Laskowski, Jabłońska, Pravda, Vařeková, & Thornton, 2018).

**Supplementary Figures**


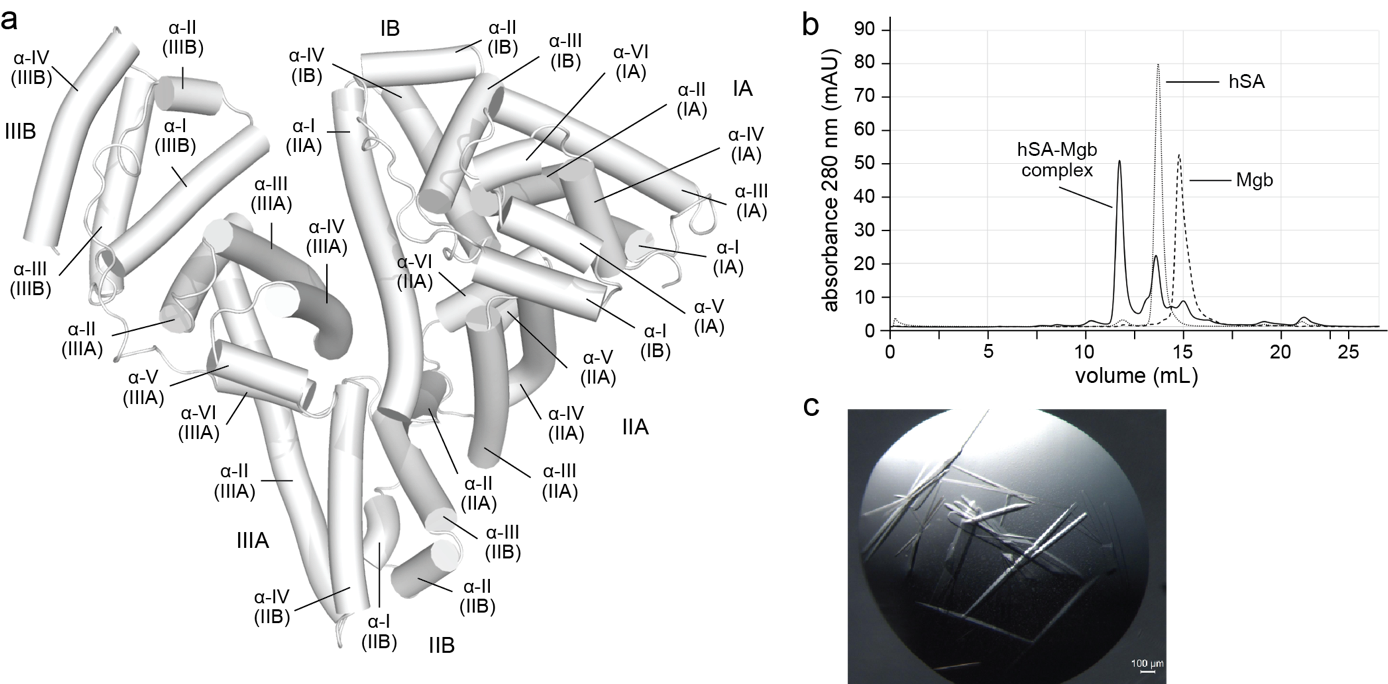


**Supplementary figure 1. a)** Structure of human serum albumin (PDB identification code: 1AO6) (Sugio, Kashima, Mochizuki, Noda, & Kobayashi, 1999). The α-helices are represented by cylinders. Each domain (I, II and III) is divided into two subdomains (A and B) formed by six and four α-helices, respectively; **b**) hSA-Mgb complex formation was confirmed by size exclusion chromatography (SEC) using a Superdex 200 10/300 GL column, equilibrated with PBS pH 7.4. hSA-Mb chromatogram is depicted by a black continuous line, while hSA and Mgb alone are depicted by small and big dots-lines, respectively. The superimposition of the chromatograms clearly confirmed the formation of hSA-Mgb complex; **c**) Representative needle crystals of hSA-Mgb complex obtained from B10 Morpheus condition in 1.2 μL drops (0.6 μL hSA-Mgb + 0.6 μL reservoir).


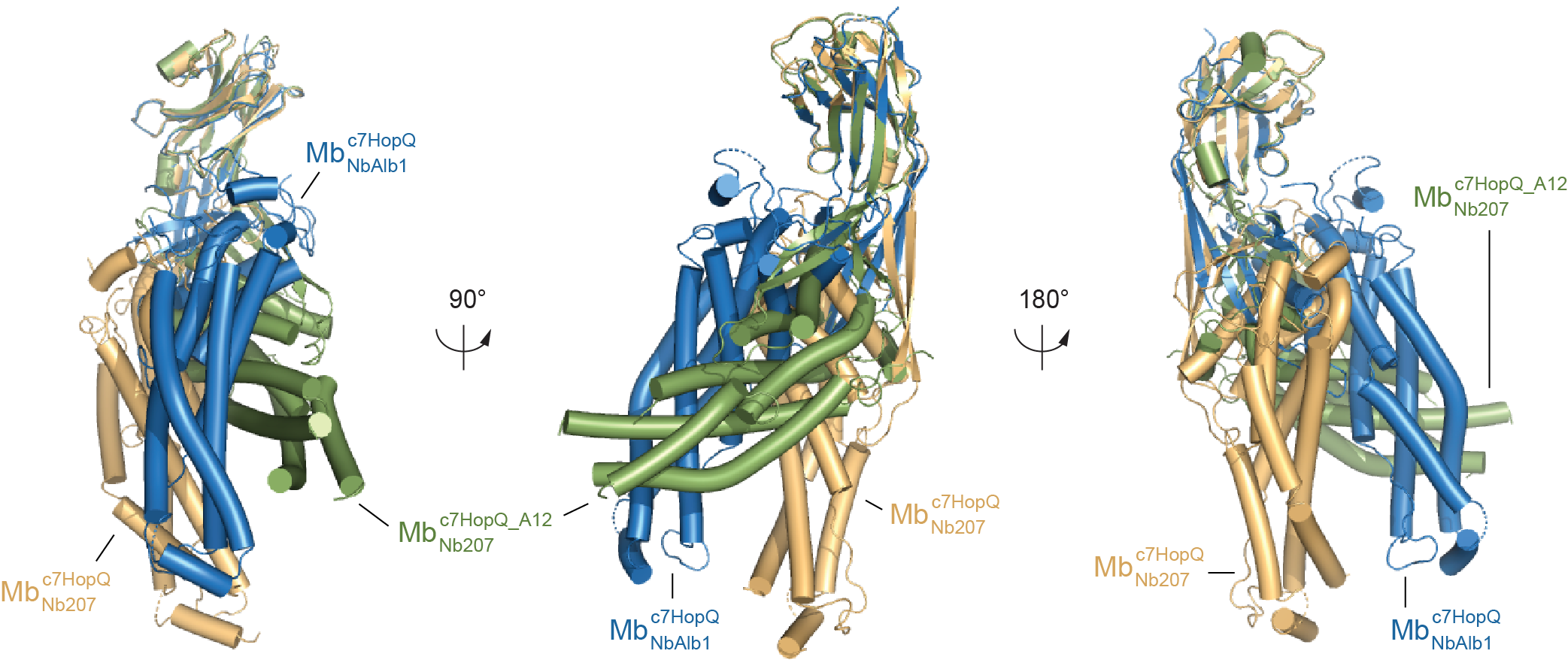


**Supplementary figure 2.** Structural comparison of different megabody fusion chimeras. Superimposition of Mb-c7HopQ-NbAlb1 used in the present study (blue; PDB identification code: 8OI2) to those designed, developed, and crystallized in the original study of Uchanski et al. (Uchański et al., 2021): Mb-Nb207-c7HopQ-A12 (light orange; PDB identification code: 6XVI) and Mb-cHopQ-Nb207 (green smudge; PDB identification code: 6QD6). Structural alignment has been performed based on the nanobody subdomain of each construct. The HopQ subdomains evidence a large plasticity, showing in the case of Mb-c7HopQ-NbAlb1 (blue) a bending by 30° in comparison to Mb-Nb207-c7HopQ-A12 (light orange) and a bending of roughly 55° followed by a torsion along its main axis if compared to Mb-cHopQ-Nb207 (green smudge).


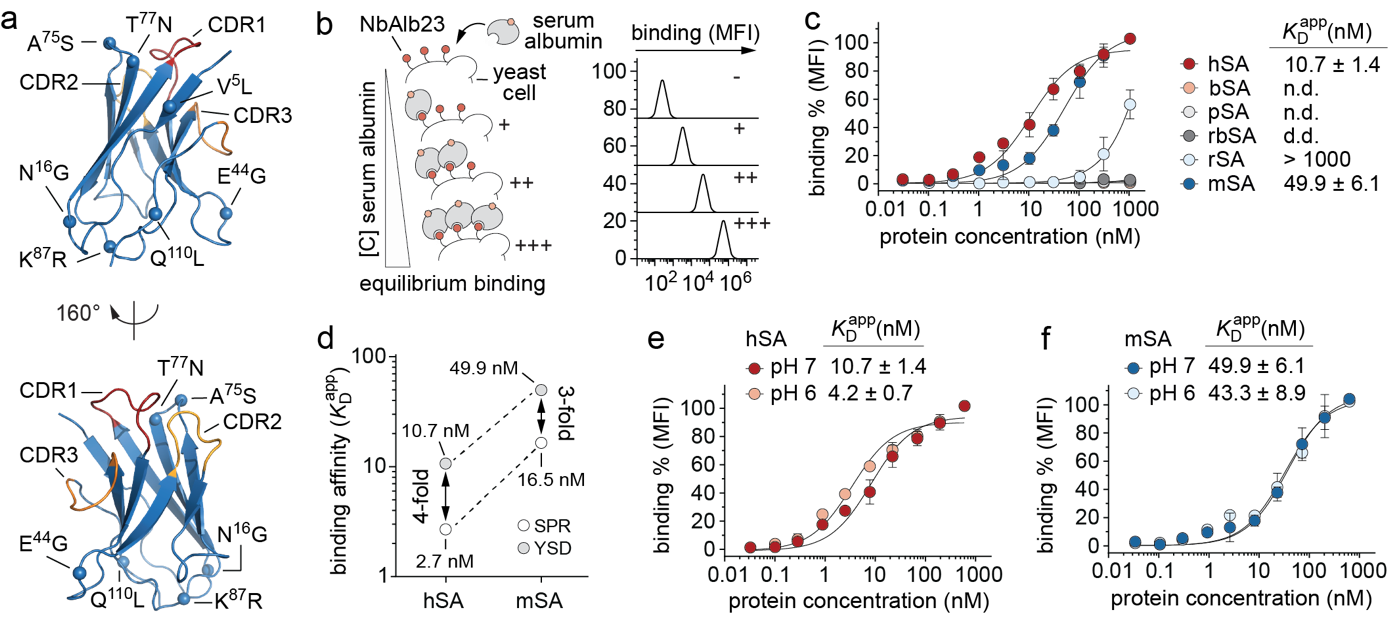


**Supplementary figure 3.** Binding characterization of humanised nanobody NbAlb23. **a)** Structure of nanobody NbAlb1 in two different orientations, with a rotation of 160°. Framework regions (FWR) are colored in skyblue, while the complementary determining regions (CDRs) are colored in firebrick (CDR1), bright orange (CDR2) and tv orange (CDR3). The $\beta$-strands are represented as arrows. Diversified amino acids acquired during the process of NbAlb1 humanization to generate NbAlb23 are depicted as spheres at the Cα positions. All mutated amino acids belonged to the FWR region. Wild type ammino acid (NbAlb1) is shown on the left of the position number while the mutated (NbAlb23) is reported on the right; **b**) Schematic representation of determination of equilibrium dissociation constants (*K*_D_) using yeast surface titration. Yeast cells expressing NbAlb23 are incubated with increasing concentrations of biotinylated SAs. The binding is reported as median fluorescence intensity (MFI) and is proportional to the amount of SAs bound to the nanobody expressed on the yeast surface; **c)** Titrations curves of the equilibrium dissociation constant of NbAlb23 towards multiple SAs determined using yeast surface display (left). The obtained *K*_D_ values expressed in nanomolar range are reported on the right. The plotted values are the results of three independent experiments and are presented as mean (dots) ± s.e.m. (bars); **d)** Comparison of *K*_D_ values of NbAlb23 against human serum albumin (hSA) and mouse serum albumin (mSA) obtained using two different and complementary techniques: surface plasmon resonance (SPR)(Patent No. WO2012175400A1, 2012)^,^ and yeast surface display; **e-f)** Titrations curves of the equilibrium dissociation constant of NbAlb23 towards hSA **(e)** and mSA **(f)** performed at acidic (pH 6) and physiological (pH 7.4) conditions.

**References**

Dombrecht, B., Peter, S., & Ververken, C. J. N. (2012). *Patent No. WO2012175400A1*. Serum albumin binding proteins.

Krissinel, E., & Henrick, K. (2007). Inference of macromolecular assemblies from crystalline state. *Journal of Molecular Biology*, *372*(3), 774–797. doi: 10.1016/j.jmb.2007.05.022.

Laskowski, R. A., Jabłońska, J., Pravda, L., Vařeková, R. S., & Thornton, J. M. (2018). PDBsum: Structural summaries of PDB entries. *Protein Science : A Publication of the Protein Society*, *27*(1), 129–134. doi: 10.1002/pro.3289

Sugio, S., Kashima, A., Mochizuki, S., Noda, M., & Kobayashi, K. (1999). Crystal structure of human serum albumin at 2.5 A resolution. *Protein Engineering*, *12*(6), 439–446. doi: 10.1093/protein/12.6.439

Uchański, T., Masiulis, S., Fischer, B., Kalichuk, V., López-Sánchez, U., Zarkadas, E., … Steyaert, J. (2021). *Megabodies expand the nanobody toolkit for protein structure determination by single-particle cryo-EM Europe PMC Funders Group*. doi: 10.1038/s41592-020-01001-6
